# Remaining Oil Distribution Law and Development Potential Analysis after Polymer Flooding Based on Reservoir Architecture in Daqing Oilfield, China

**DOI:** 10.3390/polym15092137

**Published:** 2023-04-29

**Authors:** Hongtao Fu, Zhenqiang Bai, Hu Guo, Kena Yang, Chunping Guo, Mingxi Liu, Lihao Liang, Kaoping Song

**Affiliations:** 1 Unconventional Petroleum Research Institute, China University of Petroleum (Beijing), Beijing 102249, China; 2 School of Petroleum Engineering, Northeast Petroleum University Daqing, Daqing 163318, China; 3 Research Institute of Exploration and Development of Daqing Oilfield Company Ltd., Daqing 163712, China; 4 School of Petroleum Engineering, China University of Petroleum (Beijing), Beijing 102249, China; 5 Research Institute of Petroleum Exploration and Development, Beijing 100083, China

**Keywords:** polymer flooding, reservoir architecture, remaining oil, development potential, Daqing Oilfield

## Abstract

Polymer flooding has drawn more and more attention in the world for its high incremental oil recovery factor and relative low costs compared with water flooding and other chemically enhanced oil recovery techniques. However, for many oilfields, such as Daqing Oilfield, China, that have already been flooded with polymers, how to further improve recovery remains a big problem. Traditional intralayer, interlayer and plane heterogeneity studies cannot accurately characterize the remaining oil distribution after polymer flooding. To solve this problem, we established a method to quantitatively describe the reservoir’s architecture. Then, the architecture elements were dissected hierarchically and the interface of each architecture level in Daqing Oilfield was identified. The distribution pattern and development potential of the remaining oil after polymer flooding under the influence of reservoir architecture was analyzed. The results show that, regarding the sedimentary process from north to south in Daqing Oilfield, the channel becomes narrower, the thickness decreases, the point bar’s width increases and the thickness of the meandering river decreases. The braided bar scale becomes larger and the thickness becomes smaller in the braided river. According to the reservoir’s architecture, the remaining oil was divided into four categories of plane remaining oil (abandoned channel occlusion type, interfluvial sand body occlusion type, inter-well retention type and well pattern uncontrollable type) and three types of vertical remaining oil (in-layer interlayer occlusion type, rhythm type and gravity type). About 40% of the original oil in place (OOIP) of Daqing Oilfield has not yet been produced, which indicates that there is great potential for development. This study is important for improving oil recovery in polymer-flooded reservoirs.

## 1. Introduction

**Figure 1:**
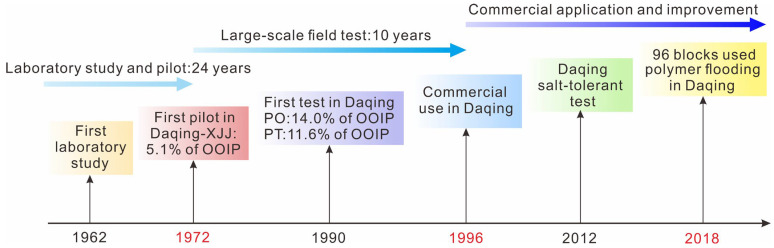
Polymer flooding development in Daqing Oilfield.

Most continental sedimentary reservoirs have shifted to the high recovery and high water-cut stages after the development of fine water injection [1,2,3]. Their crude oil production is falling rapidly. Polymer flooding is considered to be one effective technique to relieve the production decline of reservoirs [4,5,6,7]. In Daqing Oilfield, the largest continental sedimentary oilfield in China, polymer flooding technology has been tested and gradually implemented since 1972, and was commercialized in 1996. According to the latest data, 96 blocks have used polymer flooding commercially in Daqing Oilfield [8] (Figure 1). Crude oil production by polymer flooding once accounted for 23% of the total production [5]. Recent investigation has demonstrated that the remaining oil distribution after polymer flooding is more complicated and scattered, with lower injection efficiency, compared to that after water flooding [9,10]. Over time, polymer flooding has been the key area of study for remaining oil distribution, replacing water flooding [11,12,13]. In terms of oilfield development after polymer flooding, the complex reservoir architecture has taken on a significant role in determining the remaining oil distribution [14,15]. To further enhance oil recovery, in-depth studies on the impact of reservoir architecture on the remaining spatial oil distribution after polymer flooding are crucial [16,17].

Reservoir architecture is the internal geometry and spatial distribution of the reservoir. The concept was proposed by Allen in 1977, who described the geometry and internal combination of channel deposits in detail [18]. In 1983, Allen established three-level interfaces based on the Welsh Devonian sandstone [19]. On this basis, Miall extended the reservoir architecture to four-level interfaces in 1985 [20]. After continuous improvement, Miall clarified the classification method of six-level interfaces, nine architecture units and twenty lithofacies types, as in Table 1 [21]. In 1990, Weber analyzed the Fran Kine point bar architecture in Texas and established the lateral accretion model [22]. In 1991, Xue summarized the sedimentary model of the point bar and put forward the concept of a lateral accretion body for the first time [23]. Following this, many scholars differentiated the corresponding architecture units for fluvial sedimentary types and produced a large number of architecture models based on observations of modern deposits and outcrops [24,25,26,27,28,29].

Based on a detailed description of the sedimentary characteristics and superimposed relationships of the bottom bar and marginal bar, Wang further identified four types of channel sand body [30]. Li summarized the depositional patterns of reservoir architecture for different alluvial fan subfacies and concluded that it is important to clarify the structural elements of the alluvial fan for the accurate prediction of the remaining oil distribution [31]. Ovie’s research indicated that continental sedimentary reservoirs have poor lateral and vertical sand body connectivity, implying poor oil sweep efficiency and fluid injection potential [32]. Therefore, it is crucial to understand how the reservoir’s architecture controls the remaining oil distribution. This suggests that the study of reservoir architecture can accurately predict the remaining oil distribution. At present, no studies on the remaining oil distribution after polymer flooding, as related to the reservoir’s architecture, have been reported.

In this paper, we selected three typical polymer-flooded reservoirs in the process of sedimentation from the northern to the southern regions of Daqing Oilfield. Then, architecture elements were dissected hierarchically and the interface of each architecture level was identified. On this basis, the distribution law and development potential of the remaining oil after polymer flooding according to reservoir architecture in Daqing Oilfield were studied.

## 2. Reservoirs Setting

**Figure 2:**
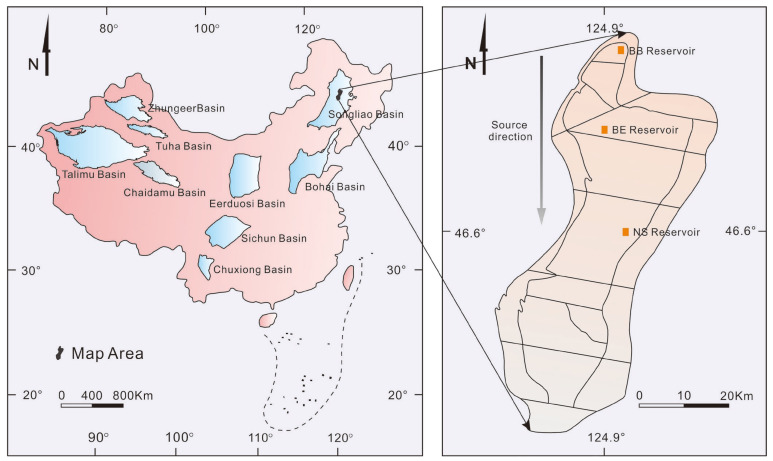
Location of different research reservoirs in Daqing Oilfield.

The Songliao basin is situated in northeast China, with a geographical position of 113–127° east longitude and 43–49° north latitude [33,34,35,36,37]. Daqing Oilfield is situated in the central depression of the first-level structural unit in the northern Songliao basin [38,39]. We selected three typical polymer-flooded reservoirs in Daqing Oilfield: BB Reservoir, BE Reservoir and NS Reservoir (Figure 2). They are located in the northern, north-central and south-central parts of Daqing Oilfield, respectively. The area of each reservoir is 3.5 km^2^, and their average permeability ranges from 500–1000 mD. To reduce the viscosity ratio and increase the swept volume to maintain a high oil production rate, the reservoirs were developed with a basic well spacing of 250 m by polymer flooding using the five-point method in 1996. The polymer flooding used polyacrylamide produced in Daqing Oilfield. For the Daqing Class-I oil layer, the main injection concentration range was 1000–1500 mg/L, the molecular weight was 2500 × 10^4^ Da and the slug size was around 0.7 PV [40]. After the polymer slugs, in 2003, water was injected. As of 2020, the total water cut of the three reservoirs had reached over 95% again.

Songliao Basin is a typical Mesozoic–Cenozoic large continental sedimentary basin [41,42,43,44,45,46]. It has undergone three main stages of tectonic evolution: (1) northern, central and southern tectonic uplift in the sedimentary stage of the Nenjiang Formation; (2) continuous southern uplift, northern and central migration at the tops of anticlines and uplift in the sedimentary stage of the Sifangtai Formation; and (3) continuous uplift leading to gradual weakening in the sedimentary stage of the Mingshui Formation [47,48,49]. Daqing Oilfield was formed in the late stage of the Mingshui Formation in the central depression area, with a SN-trending distribution. The source bed, reservoir stratum and cap rock of Daqing Oilfield belong to the Cretaceous strata of Qingshankou Formation, Yaojia Formation and Nenjiang Formation [50,51].

**Figure 3:**
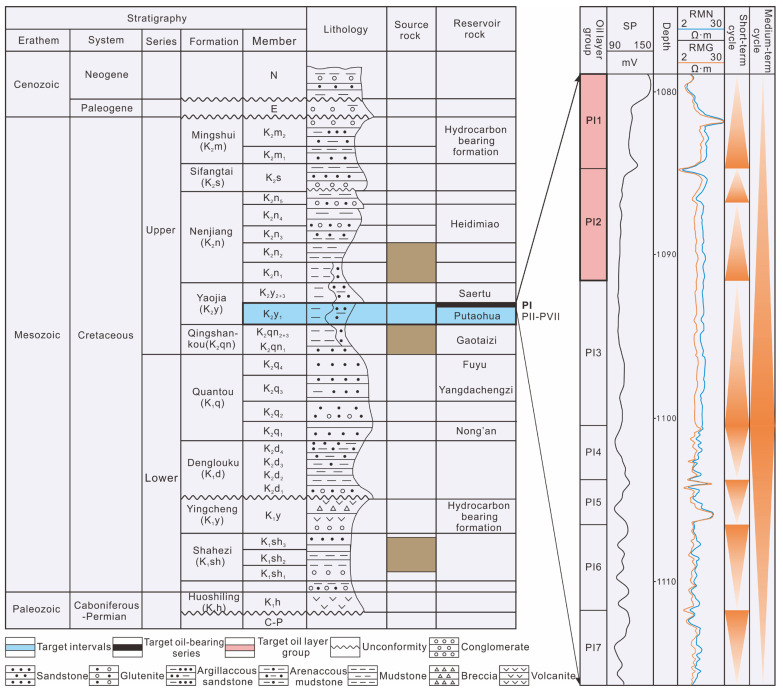
Stratigraphy and evolution of Songliao Basin and stratigraphic column of X well.

The reservoir of Daqing Oilfield was formed by fluvial–deltaic sedimentary clastic rocks in inland lake basins of the Lower Cretaceous [38,39]. Lake aggression and regression at different scales occurred many times in the sedimentary process, forming many sand bodies with different facies zones, different scales and different physical properties. These sand bodies were interspersed and intertwined with each other longitudinally, and the various facies zones changed frequently in the plane. This formed a very complex sedimentary reservoir system, with significant reservoir heterogeneity [52]. At present, the developed target formations are known as Saertu (S), Putaohua (P) and Gaotaizi (G). Dozens to hundreds of sedimentary units with different properties can be drilled in a single well [53]. From the cores of these sedimentary units, it can be observed that the thickness of the sand body varies from a few centimeters to more than a dozen meters. The P oil formation is divided into seven sedimentary units, including PI-PVII. The target layer in this study is the PI1-PI2 sedimentary unit (Figure 3).

## 3. Characterization Methods of Reservoir Architecture

### 3.1. Single-Channel Division and Internal Architecture

**Figure 4:**
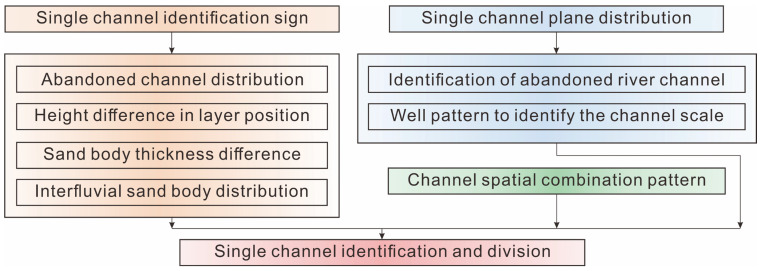
Identification process of a single channel in a meandering river.

The single channel sand body division is mainly for the meandering river compound channel sand body [54,55,56,57,58,59,60]. First, according to the theoretical model of single-channel deposition of meandering rivers and the empirical equation of channel scale, the single-channel sand body’s scale was determined [18,19,61,62]. Then, various single-channel identification marks were applied (I: abandoned channel distribution; II: height difference in layer position; III: sand body thickness difference; IV: interfluvial sand body distribution) in order for the well-connected profile to determine the distribution of the single-channel profiles. Abandoned channels and infilled well patterns were used to determine the single-channel sand body scale and the plane distribution. Finally, the identification and division of the single-channel sand body in 3D space were completed by spatially combining the single channel (Figure 4).

The spatial combination model of the single-channel sand body was utilized in a compound-channel sand body according to the vertical and plane distribution, as well as to the infill well pattern data.

(1) Superposition of single-channel sand bodies in different periods in the same sedimentary unit.

**Figure 5:**
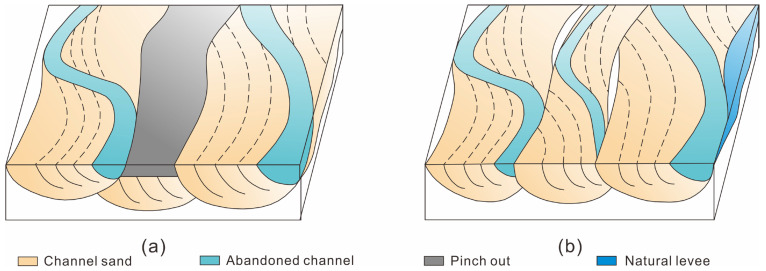
Spatial combination model of a single channel of a meandering river. (**a**) Superposition of single-channel sand bodies in different periods in the same sedimentary unit, (**b**) simultaneous superposition of single-channel sand bodies within the same sedimentary unit.

According to the single-channel sand body identification marker, there are differences in the elevation of top surface and sand body scales for each single channel in this model. These varying sand bodies can create local lateral assemblies at various time points (Figure 5a).

(2) Simultaneous superposition of single-channel sand bodies within the same sedimentary unit.

In this model, there is little difference in elevation between individual channels, and the channels form at the same time in different locations. Overbank sand bodies, flood plains and the last stages of abandoned channel deposits exist between the single channels (Figure 5b).

### 3.2. Internal Architecture of Point Bar and Braided Bar

#### 3.2.1. Internal Architecture of Point Bar

For the identification of the point bar and braided bar, their scales were calculated by categorizing the plane distribution of the channel into abandoned channel and permeability attributes. The empirical equation for the scale of the point bar and braided bar, summarized by a modern deposit, was used to determine the plane distribution of the point bar and braided bar sand bodies [61,63]. Then, the vertical sequences and curve shapes of the point bar and braided bar sand bodies were used, combining their developmental scales and determining the plane distribution.

**Figure 6:**
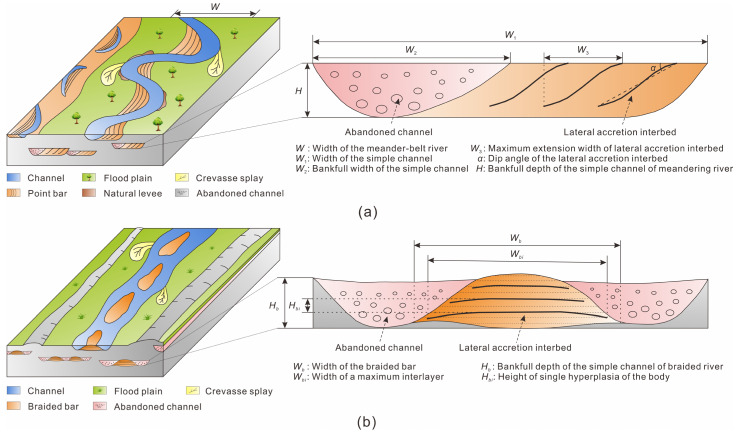
Sand body morphology and relevant parameters. (**a**) Internal architecture of point bar, (**b**) internal architecture braided bar.

The identification of inner structures of the point bar and braided bar was based on the modern deposit theory and outcrop observation [39,64]. We performed reservoir architecture prediction with dense well pattern data. A quantitative distribution model for aspects of internal architecture, such as the scale and occurrence of lateral accretion in the interbed of the point bar sand body and braided bar sand body, was built [65]. It is important to note that in order to fully demonstrate the morphology of lateral accretion interbed, the architecture model was divided into a plane grid of 5 m × 5 m × 0.1 m. The scale, direction, extension length, horizontal spacing and dip angle of the lateral accretion interbed in the point bar were determined by the following methods:

(1) Scale of point bar: after determining the width of the meander-belt river or the bankfull depth of the simple channel, the bankfull width of the channel was calculated with an empirical equation (Equation (1) or (2)) [66] (Figure 6a). Then, the length of the point bar were calculated using the following equation [25]:

(1) $$W=7.44W_{2}{}^{1.01}$$

(2) $$\log W_{2}=1.54\log H+0.83$$

(3) $$L_{p}=0.8531\ln(W_{2})+2.4531$$

where *W* is the width of the meander-belt river, m; *W*_2_ is the bankfull width of the simple channel, m; *H* is the bankfull depth of the simple channel of meandering river, m; and *L_p_* is the length of the point bar, m.

(2) Direction of lateral accretion interbed: lateral accretion interbed in the point bar faced the concave bank of an abandoned channel, and also inclined in the direction of the abandoned channel [67].

(3) Maximum extension width of lateral accretion interbed: The maximum extension width of the lateral accretion interbed was about 2/3 of the full bank width of the channel (Equation (4)) [66].

(4) $$W_{3}=2W_{2}/3$$

where *W*_3_ is the maximum extension width of the lateral accretion interbed, m.

**Figure 7:**
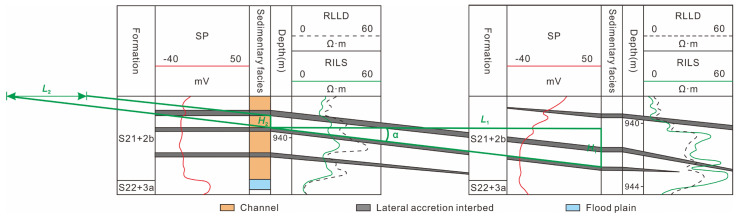
Distribution of internal lateral accretion interbed of the point bar.

(4) Dip angle of lateral accretion interbed: the dip angle of the lateral accretion interbed was calculated by an empirical equation using small well spacing in the point bar sand body [68] (Figure 7). The computational equation of the dip angle is shown below [66]:

(5) $$\tan\alpha =H_{1}/L_{1}$$

where *α* is the dip angle of the lateral accretion interbed, in degrees; *H*_1_ is the relative elevation difference between two wells on the same lateral accretion interbed, m; and *L*_1_ is the distance between two wells, m.

(5) Horizontal spacing of lateral accretion interbeds: the lateral accretion interbed connecting two wells with small well spacing extended along the lateral accretion direction and intersected with the top surface of the point bar [15]. The projected distance on the plane between the intersection points of adjacent lateral accretion interbeds was the horizontal spacing of lateral accretion interbeds, *L*_2_ (Figure 7).

(6) $$L_{2}=H_{2}/\tan\alpha$$

where *L*_2_ is the horizontal spacing of lateral accretion interbeds, m; *H*_2_ is the height of the lateral accretion body, m.

#### 3.2.2. Internal Architecture Braided Bar

The interlayer dip angle in the center of the braided bar was less than 2° [38,69], showing an approximately horizontal distribution. Therefore, the interbed dip angle in the braided bar will not be discussed. The length and width of the interlayer of the braided bar’s lateral accretion were determined by the following methods.

(1) The relationship between the width of the braided bar *W_b_* and the height of the braided bar *H_b_*, and that between the length of the braided bar *L_b_* and the width of the braided bar (Figure 6b) *W_b_*, were calculated by the following empirical equation [70]:

(7) $$W_{b}=11.413H_{b}{}^{1.4182}$$

(8) $$L_{b}=4.9517W_{b}{}^{0.9676}$$

where *W_b_* is the width of the braided bar, m; *H_b_* is the bankfull depth of the simple channel of braided river, m; and *L_b_* is the length of the braided bar, m.

(2) The developmental scale of the interlayer in the braided bar was controlled by the scale of single hyperplasia of the body; the maximum width of the interlayer was the width of single hyperplasia of the body, and the maximum length was that of single hyperplasia of the body [71]. The maximum width of the interlayer *W_b__i_* was calculated by using an empirical equation (Equation (9)) [70]:

(9) $$W_{bi}=15.954H_{bi}{}^{1.3723}$$

where *W_bi_* is the maximum width of the interlayer, m; and *H_bi_* is the height of single hyperplasia of the body.

#### 3.2.3. Geological Modeling of Reservoir Architecture

**Figure 8:**
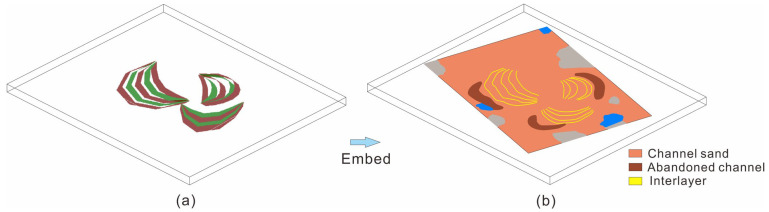
Lateral accretion interbeds are embedded in the geological model. (**a**) Lateral accretion interbeds distribution patterns. (**b**) spatial distribution of the point bar’s internal lateral accretion interbed.

The internal architecture of a reservoir is a separate property [71,72,73,74,75]. In this paper, we used spatiotemporal sequential indication simulation to deal with discrete variables [61]. It is worth noting that the simulation was not able to accurately characterize the exact complex spatial morphology of the point bar sand body in aspects such as internal configuration [76,77]. Therefore, the model was reprocessed using human–computer interaction method for the lateral interlayer inside the point bar, so that the spatial form of the lateral interlayer conformed to the practical understanding of geology. Finally, a 3D geological model of the lateral interlayer was established according to the dissection results of the point bar sand body’s internal architecture (dip, extension length, dip angle and horizontal spacing of lateral accretion interbed). Lateral accretion interbeds between profiles were connected in crescent distribution patterns (Figure 8a). The lateral accretion interbed model was embedded into the 3D microfacies model [60]. The spatial distribution characteristics of the point bar’s internal lateral accretion interbed are displayed in Figure 8b. This method could also be used in internal architecture analyses of the braided river sand body, the underwater distributary channel sand body and the 3D space characterization of internal interfaces, at all levels.

## 4. Results and Discussion

### 4.1. Quantitative Distribution of Reservoir Architecture Elements

#### 4.1.1. BB Reservoir

**Figure 9:**
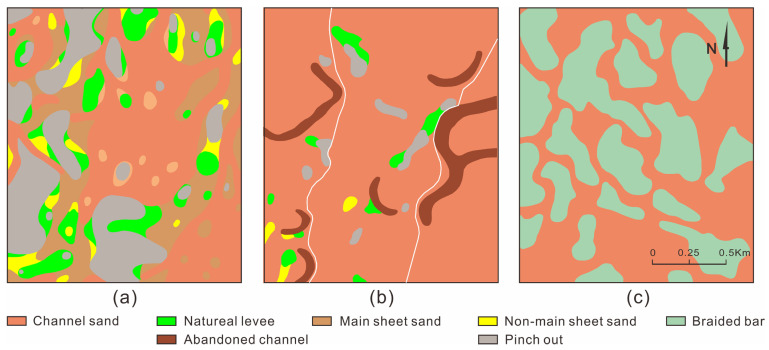
Sedimentary facies of different units in BB Reservoir. (**a**) PI1 sedimentation unit, (**b**) PI21 sedimentation unit, (**c**) PI122 sedimentation unit.

The PI1 unit was straight underwater distributary channel deposition. The channel sand body was distributed in thin strips and small lumps at a small scale. The simple channel sand body’s width was 100–150 m, and its thickness was 2–4 m. Sheet sand was poorly developed, and off-surface sand and pinch-out were more developed (Figure 9a). The channel sand body of the PI21 unit was distributed in a continuous sheet, with scattered overflow sediment. The width of the simple channel sand body was 700–1200 m, and its thickness was 5–6 m. The point bar sand body was developed with a width of 100–150 m and a thickness of 6–8 m (Figure 9b). The PI122 unit was adjacent to the delta deposition area, and the channels were distributed in a continuous sheet. The channel sand body width of the braided river was greater than 1500 m, and the thickness was 5–7 m. The braided bar sand body was developed with a longitudinal length of 100–500 m, a width of 100–400 m and a thickness of 6–9 m (Figure 9c).

#### 4.1.2. BE Reservoir

**Figure 10:**
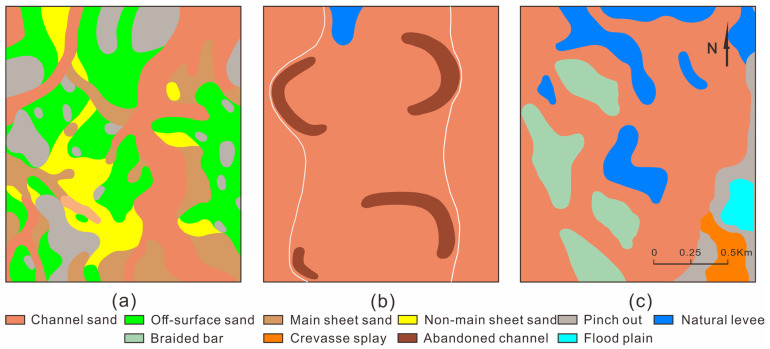
Sedimentary facies of different units in the BE Reservoir. (**a**) PI1 sedimentation unit, (**b**) PI21 sedimentation unit, (**c**) PI231 sedimentation unit.

The PI1 unit was straight upper-river channel deposition. The channel sand body was distributed in interwoven strips at a large scale. The simple channel sand body’s width was 200–500 m, and the thickness was 2.5–4 m. The sheet sand developed poorly, while the off-surface sand and pinch-out were more developed (Figure 10a). The channel sand body of the PI21 unit was distributed in a continuous sheet, with scattered overflow sediment. The width of the simple channel sand body was 600–1000 m, and the thickness was 5.5–7 m. The point bar sand body was well-developed, with a width of 250–350 m and a thickness of 5.5–7 m (Figure 10b). The braided river was located to the west of the PI231 unit, and the width of the braided river’s channel sand body was greater than 1500 m. The braided bar sand body was well-developed, with a longitudinal length of 300–800 m, a width of 150–300 m and a thickness of 5.5–7 m. The flood deposit area was located to the east of the braided river, where the main developments were natural levees, crevasse splays and flood plain deposits (Figure 10c).

#### 4.1.3. NS Reservoir

**Figure 11:**
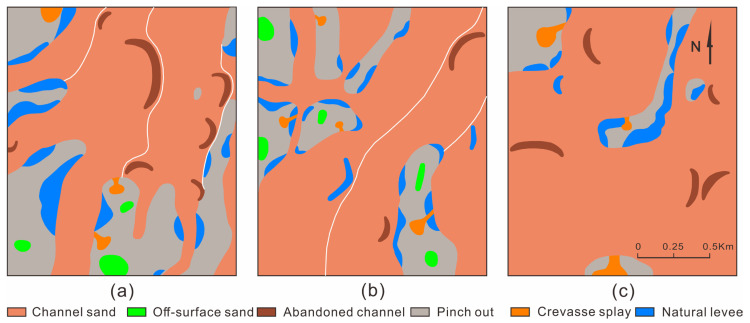
Sedimentary facies of different units in the NS Reservoir. (**a**) PI1b sedimentation unit, (**b**) PI2b sedimentation unit, (**c**) PI2c sedimentation unit.

The channel sand body of the PI1b unit was distributed in a continuous sheet, the pinch-out was relatively developed and the off-surface sand was sporadically developed. The width of the simple channel sand body was 300–500 m, and the thickness was 3–5 m. The point bar sand body was developed, with a width of 350–400 m and a thickness of 4–5 m (Figure 11a). The channel sand body of the PI2b unit was distributed in a continuous sheet, the pinch-out was relatively developed, and the off-surface sand was sporadically developed. The width of the simple channel sand body was 350–400 m, and the thickness was 5–6 m. The point bar sand body was well-developed, with a width of 400–600 m and a thickness of 5–6 m (Figure 11b). The channel sand body of the PI2c unit was distributed in a continuous sheet, the pinch-out was relatively developed, and the off-surface sand was sporadically developed. The width of the simple channel sand body was 400–500 m, and the thickness was 3–5 m. The point bar sand body was well-developed, with a width of 400–500 m and a thickness of 4–6 m (Figure 11c).

In the sedimentary process from north to south in the Daqing Oilfield, the upper-river channel evolves into an underwater distributary channel, the thicknesses of the channels gradually decrease, and the shapes of the channels change from wide interwoven strips to narrow strips. The PI2 unit was divided into the PI21 and PI22 units in the BB Reservoir. The braided river of the PI22 unit runs southward into the braided river of the PI231 and PI232 units. The channel’s morphology changes from bifurcated and interwoven strips to wide strips, and the thicknesses of the channels gradually decrease. As the meandering river of the PI21 unit runs southward, the widths of the channels gradually narrow from 700–1200 m to 300–500 m, and the thicknesses of the channels gradually decrease. From the BB Reservoir to the NS Reservoir, the widths of the point bars gradually increase from 100–150 m to 400–600 m, and the thicknesses of the point bars gradually decrease from 6–8 m to 4–5 m. From the BB Reservoir to the western BE Reservoir, the lengths of braided bars incearse from 100–500 m to 300–800 m, the thickness becomes smaller, and the scale of the overall braided bar becomes larger, but the number of braided bars decreases.

In the sedimentary process from north to south in the Daqing Oilfield, both the numbers and thicknesses of in-layer interlayers decrease. The interlayers of the braided rivers are mostly horizontal and scattered. The dip angle of the interlayer in a straight river is mostly horizontal, the interlayer extension along the flow direction is generally greater than 250 m, and the scale of vertical interlayer extension relative to the flow direction is controlled by the width of the channel [78]. In the sedimentary process from north to south, the scale of the single channels gradually decreases. The dip angle of the in-layer interlayer tended to increase in the sedimentary process from north to south, but the overall dip angle was less than 7° (Table 2).

### 4.2. Influence of Reservoir Architecture on Remaining Oil

In order to prevent coring from being polluted by drilling fluid, and to accurately determine the displacement efficiency of the oil layer, closed coring was performed in Daqing Oilfield for major polymer-flooded reservoirs from 2004 to 2008. According to the classification standard of displacement efficiency in Daqing Oilfield, the displacement efficiency of the core in pressured coring wells was divided into four categories: high displacement efficiency (HDE), medium displacement efficiency (MDE), low displacement efficiency (LDE) and no displacement efficiency (NDE). The displacement efficiency ranges are given in Table 3.

For sand bodies in straight channels, the interlayer extension along the flow direction is generally greater than 250 m. The scale of vertical interlayer extension relative to the flow direction is controlled by the channel’s width. The channel sand body’s scale is narrow and small. There is remaining gravity-type oil at the top of the reservoir after polymer flooding. Sand bodies are connected between the upper and lower single layers where there is no interlayer in the braided river sand body. Polymer flooding is affected by gravity and the rhythm of replacing the lower high-permeability oil layer (MDE or HDE), so the remaining oil in the upper layer (LDE or NDE) is rich. The interlayer in the braided river sand body acts as a vertical flow barrier, and the polymer flooding effect is good in the upper and lower reservoirs (MDE or HDE). There is, essentially, no remaining rich oil. In the area of the abandoned channel with poor occlusion and connectivity, between the single-channel sand bodies of the meandering river, remaining oil is collected at the edge of the channel after polymer flooding. In addition, the remaining oil is relatively rich where there is lower channel connectivity thickness, at the edge of the channel.

**Figure 12:**
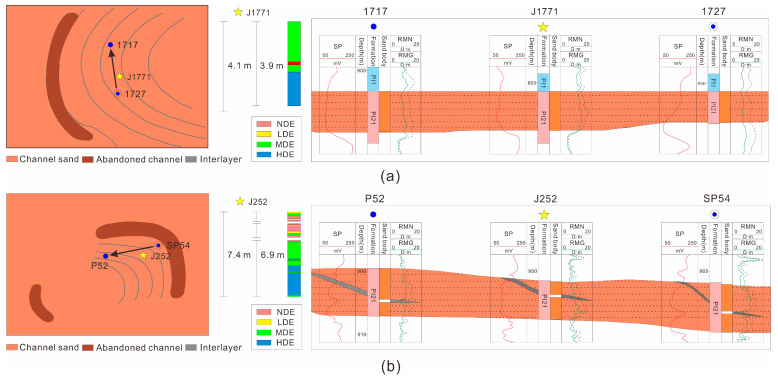
Injection–production relationship in point bars. (**a**) Fluid is injected along the direction of the interlayer, (**b**) fluid is injected vertically relative to the direction of the interlayer.

It is worth noting that the point bar sand body of the meandering river usually exhibits significant internal heterogeneity. As it is affected by the relationship between the interlayer and the injection–production well, the remaining oil distribution is different in different directions. The results of the displacement efficiency distribution from the closed coring interval show that if fluid is injected along the direction of the interlayer in the meandering river’s point bar sand body, the polymer-flooded thickness ratio is more than 90%. The thickness ratio of HDE reaches 39.2%, and the thickness ratio of MDE reaches 57.1% (Figure 12a). The fluid cannot be blocked by the interlayer between injection–production wells; thus, a the reservoir is produced, and there is less remaining oil after polymer flooding.

If fluid is injected vertically relative to the direction of the interlayer in the meandering river’s point bar sand body, the polymer-flooded thickness ratio is only 76.2%. The thickness ratio of HDE is 38.8%, and the thickness ratio of MDE is 35.8%. The injected fluid can obviously be blocked by the lateral accretion interbed, leading to the reservoir being poorly produced, and there is more remaining oil after polymer flooding (Figure 12b). In addition, as the in-layer interlayer dip angle tends to increase along with the sedimentary process from north to south, at the same developmental scale, the shielding effect on the remaining oil is relatively stronger.

### 4.3. Remaining Oil Distribution Law

**Figure 13:**
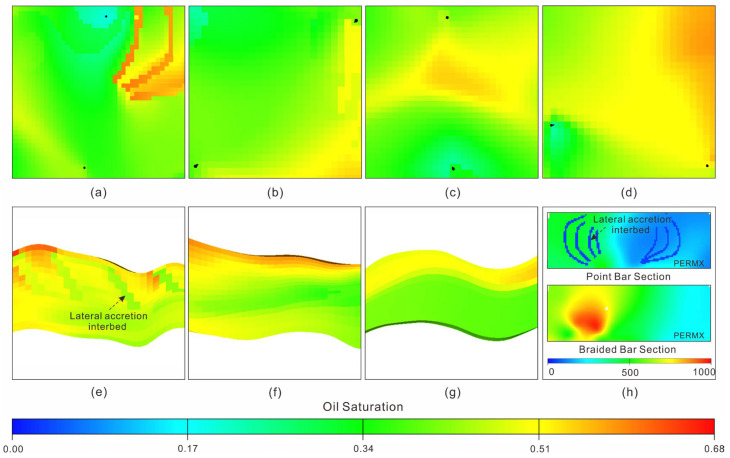
Remaining oil distribution of fluvial sand body after polymer flooding. (**a**) Abandoned channel occlusion type, (**b**) interfluvial sand body occlusion type, (**c**) interwell retention type, (**d**) well pattern uncontrollable type, (**e**) in-layer interlayer occlusion type, (**f**) rhythm type, (**g**) gravity type, (**h**) permeability sections the X direction of point bar and braided bar.

In order to reduce the amount of simulation calculations, we coarsened the grids and property data and imported them into the Eclipse black-oil model for historical fitting of the fixed production fluid combined with the determined dynamic data [79]. During the process of historical fitting, the fitting errors of the reserves in each reservoir were less than 5%; the water cut fitting degree of each typical reservoir was more than 80% (with month as the time node, water cut error = (actual water cut − calculated water cut)/actual water cut; when water cut is ≤1.0%, it is the standard and compliant water cut time node; water cut fitting degree = standard and compliant water cut time nodes/all water cut time nodes). When the historical fitting met the above standard, the remaining oil after polymer flooding was analyzed by taking into account the reservoir’s architecture (Figure 13h). According to the detailed dissection results of the reservoir’s internal architecture, two main factors affecting the genesis of the remaining oil, namely, geology and development, were determined. Four remaining planar oil distribution types (abandoned channel occlusion type (Figure 13a), interfluvial sand body occlusion type (Figure 13b), interwell retention type (Figure 13c) and well pattern uncontrollable type (Figure 13d)) and three remaining vertical oil distribution types (in-layer interlayer occlusion type (Figure 13e), rhythm type (Figure 13f) and gravity type (Figure 13g) were classified (Table 4).

For the remaining oil distribution after polymer flooding in the braided river sand body, the gravity type accounted for more than 30%, followed by the occlusion type and rhythm type, and the proportion of the well pattern imperfect type was only about 10%. From the perspective of a single factor, the quantities of remaining oil after polymer flooding were in the following order: gravity type, rhythm type, abandoned channel occlusion type, well pattern uncontrollable type, interfluvial sand body occlusion type and in-layer interlayer occlusion type. For the remaining oil distribution after polymer flooding in the meandering river sand body, the occlusive type accounted for more than 45%, follow by the gravity type and rhythm type, and the proportion of the well pattern imperfect type was less than 17%. From the perspective of a single factor, the quantities of remaining oil after polymer flooding were in the following order: gravity type, rhythm type, abandoned channel occlusion type, in-layer interlayer occlusion type, interfluvial sand body occlusion type, well pattern uncontrolled type and interwell retention type (Table 5).

### 4.4. Development Potential Analysis

**Figure 14:**
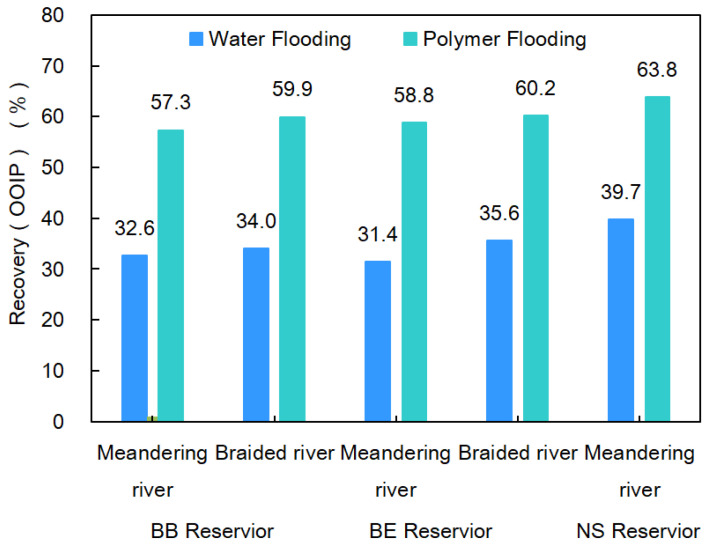
Histogram of meandering river and braided river recoveries in each reservoir.

The BB Reservoir, BE Reservoir and NS Reservoir are located from the north to the south of Daqing Oilfield. For the BB Reservoir, the braided river sand body accounts for 14.4% of the total reserves and the meandering river sand body accounts for 85.6%. For the BE Reservoir, the braided river sand body accounts for 11.1% of the total reserves and the meandering river sand body accounts for 88.9%. In the NS Reservoir, there is only a meandering river sand body. When the water cut reached 95% after water flooding, the recovery of the BB Reservoir’s braided river sand body was 34.0%, and that of the meandering river sand body was 32.6%. The recovery of the BE Reservoir’s braided river sand body was 35.6% and that of meandering river sand body was 31.4%. The recovery of the NS Reservoir’s meandering river sand body was 39.7%. Water flooding was initiated following the injection of 0.7PV polymer into the three reservoirs. When the water cut reached 95% again, the recovery of the braided river sand body was 59.9%, and that of the meandering river sand body was 57.3% in the BB Reservoir. For the BE Reservoir, the recoveries for the braided river sand body and the meandering river sand body were 60.2% and 58.8%, respectively. For the NS Reservoir, the meandering river recovery was 63.8%. The above values indicate that when the meandering river sand body and the braided river sand body were developed in the same oil-bearing layer, the recovery capability of the braided river sand body after water flooding or polymer flooding was higher. With the process of meandering river deposition, the recovery of the sand body after polymer flooding increased gradually. The reserves of Daqing Oilfield still have about 40% potential after polymer flooding, as shown in Figure 14.

## 5. Conclusions

(1) We quantitatively characterized the architectural elements of fluvial sedimentary reservoirs, and their variation was found. In the sedimentary process from north to south in Daqing Oilfield, the simple channel width becomes narrower, and the thickness of the meandering river decreases. The point bar sand body’s width becomes larger, the thickness decreases, and the dip angle of the interlayer inside the point bar sand body becomes smaller. The braided bar sand body’s scale becomes larger, the thickness decreases, and the number of braided bar sand bodies decreases in braided rivers.
(2) Sand bodies are connected between the upper and lower single layers in the area where there is no interlayer present in the braided river sand body. Polymer flooding is affected by gravity and rhythm and replaces the lower high-permeability oil layer; the remaining oil is rich in the upper oil layer. In the area with poor occlusion and connectivity of the abandoned channel in the single-channel sand body of the meandering river, the remaining oil is located at the edge of channel after polymer flooding.
(3) According to the detailed dissection results of the reservoir architecture, four remaining planar oil distribution types (abandoned channel occlusion type, interfluvial sand body occlusion type, interwell retention type and well pattern uncontrollable type) and three remaining vertical oil distribution types (in-layer interlayer occlusion type, rhythm type and gravity type) were classified. For the remaining oil distribution types, the proportion of the gravity type was more than 30% in the braided river sand body, and the proportion of the occlusion type was more than 40% in the meandering river sand body.
(4) After polymer flooding, Daqing Oilfield still has about 40% OOIP, and the remaining oil reserves have high development potential. The development measures of remaining oil in Daqing Oilfield from north to south should transform from the method of expanding the sweep volume to the method of improving the displacement efficiency.

## Figures and Tables

**Table 1:** Fluvial Sedimentary Reservoir Architecture Classification (Revised after Miall [21]).

Architecture Interface Level	Architecture Units	Time Scale (a)	Examples of Sedimentation	Reservoir Correlation Unit Classification
Level 0	Lamina	10^−6^	Small-scale ripple mark	/
Level 1	Microforms	10^−5^–10^−4^	Large-scale ripple mark	/
Level 2	Mesoforms	10^−2^–10^−1^	Dunes	/
Level 3	Macroforms accreted terrace	10^0^–10^1^	Lateral accretion body	/
Level 4	Macroforms	10^2^–10^3^	Point bar, natural levee, crevasse splay	Single layer (sedimentary unit)
Level 5	River channel	10^3^–10^4^	Single channel	Single layer
Level 6	River channel zone	10^4^–10^5^	Composite channel	Sands group
Level 7	Large sedimentary system	10^5^–10^6^	Level 4 cycle (Milankovitch)	Oil layer group
Level 8	Basin-fill complex	10^6^–10^7^	Level 3 cycle (Milankovitch)	Oil-bearing series

**Table 2:** Quantitative distribution law of meandering river architecture elements in different reservoirs.

Reservoirs	Sedimentary Unit	Width of Simple Channel Sand Body (m)	Width of Point Bar Sand Body (m)	Thickness of Point Bar Sand Body (m)	Dip Angle of Lateral Accretion Interbed (°)
BB Reservoir	PI21	700–1200	100–150	6–8	4
BE Reservoir	PI21	600–1000	150–350	5.5–7	5
NS Reservoir	PI1b	300–500	350–400	4–5	5
	PI2b	350–400	400–600	5–6	7
	PI2c	400–500	400–500	4–6	5

**Table 3:** Classification criteria of displacement efficiency.

Type	High Displacement Efficiency (HDE)	Medium Displacement Efficiency (MDE)	Low Displacement Efficiency (LDE)	No Displacement Efficiency (NDE)
value (%)	>55	35–55	15–35	<15

**Table 4:** Remaining oil distribution types of fluvial sand bodies after polymer flooding.

Types	Distribution Type of Remaining Oil	Characteristics of Remaining Oil Distribution after Polymer Flooding
Planar	Abandoned channel occlusion type	Blocked by the abandoned channel, the remaining oil is rich at the edge of abandoned channel [15].
	Interfluvial sand body occlusion type	The displacement fluid advances along the channel center, and the remaining oil is rich at the edge of the channel sand body [30].
	Interwell retention type	In the well pattern, the displacement fluid advances along both sides of main flow line, and the remaining oil is rich at the diverting flow line [80].
	Well pattern uncontrollable type	The reservoir sand body is widely distributed, and remaining oil uncontrolled by the well pattern is formed at its edge or extension area, with poor physical properties [15].
Vertical	In-layer interlayer occlusion type	In the meandering river sand body, the remaining oil is rich in lateral accretion interbeds due to the occlusion of the lateral accretion interbed [15].
	Rhythm type	The rhythm distribution of thick reservoirs is complex, and the remaining oil is rich under the action of positive rhythm, reverse rhythm and compound rhythm [81].
	Gravity type	Due to the influence of gravity, the degree of water flooding in the lower part of the thick reservoir is much higher than that in the upper part, and the remaining oil is rich in the upper part [82].

**Table 5:** Proportion of remaining oil and distribution patterns in the meandering river and braided river of each reservoir.

Reservior	Sand Body Type	Injection–Production Imperfect Type (%)		Occlusion Type (%)			Rhythm Type (%)	Gravity Type (%)
		Interwell Retention Type	Well Pattern Uncontrolled Type	Abandoned/Braided Channel	Interfluvial Sand Body	In-layer Interlayer		
BB Reservior	Meandering river	3.0	6.0	22.0	11.0	13.0	21.6	23.4
	Braided river	6.0	7.0	20.0	6.0	2.0	28.3	30.7
BE Reservior	Meandering river	6.0	8.0	10.0	15.0	19.0	16.8	25.2
	Braided river	11.0	9.0	12.0	10.0	3.0	22.0	33.0
NS Reservior	Meandering river	2.0	6.0	13.0	14.0	18.0	9.4	37.6

## Data Availability

Not applicable.
